# Effect of uterine torsion intrapartum on concentrations of placental estrogens and progesterone in cattle

**DOI:** 10.1002/vms3.1145

**Published:** 2023-06-19

**Authors:** Sait Sendag, Marlene Sickinger, Talha Arslan, Gerhard Schuler, Axel Wehrend

**Affiliations:** ^1^ Clinic for Obstetrics and Gynecology Veterinary Medicine Van YYÜ Van Turkey; ^2^ Clinic for Ruminants (Internal Medicine and Surgery) Faculty of Veterinary Medicine Justus‐Liebig‐University Giessen Germany; ^3^ Department of Econometrics Van YYÜ Van Turkey; ^4^ Clinic for Obstetrics, Gynecology and Andrology of Large and Small Animals with Ambulatory Service Faculty of Veterinary Medicine Justus‐Liebig‐University Giessen Germany

**Keywords:** cow, intrapartum blood progesterone, placental estrogens, uterine torsion

## Abstract

**Objective:**

The current study investigates how uterine torsion influences placental oestrogens and progesterone blood concentrations in intrapartum cows. Our research tests the hypothesis that intrapartum uterine torsion impairs the ability of the placenta to synthesize steroids and may also suppress the release of synthesized steroids into the maternal circulation.

**Methods:**

The study included a total number of 37 intrapartum dairy cows of various breeds and ages. These animals were transported to our clinic by their owners. Furthermore, general and obstetrical examinations of all these animals were performed in our clinic. The uterine torsion (UT) group consisted of 20 animals. The presence of UT was verified during clinical general examinations by vaginal and transrectal examination. The comparison (C) group included 17 animals whose birth was undisturbed or could be terminated with moderate obstetrical assistance. The clinical examination of group C animals showed no problems with their general health and genital organs. Blood samples were collected immediately after the initial obstetrical examination from 37 cows for radioimmunological measurement of estradiol‐17β (E2), free total estrogen (FTE), conjugated total estrogen (CTE), and progesterone (P4).

**Results:**

In terms of P4, there was no statistical difference between the two groups. For all estrogen parameters, however, concentrations were significantly lower in the UT group than in the C group. In the correlation analysis, there was a significant correlation between the P4 and the FTE in the C group. Furthermore, the positive correlation between all estrogen parameters in the UT group was significant. In group C, significant positive correlations were found apart from the correlation between E2 and CTE.

**Conclusions:**

The results are consistent with the hypothesis and suggest that in UT animals processes dependent on estrogens or other placental hormones may be impaired during the peri‐ or postpartum period.

## Abstract 2

The current study's findings confirm that intrapartum UT in cows clearly has an adverse influence on the production of placental estrogens and/or on their release into the maternal circulation, considerably decreasing peripheral levels of estrogen.

## 1 INTRODUCTION

In the cow, the morphological and functional peripartum changes, such as cervical dilation and uterine contractions, are regulated to a significant extent by endocrinological interactions (Burton et al., 1987; Kindahl et al., 2002; Smith et al., 1973; Wehrend, 2003). Steroid hormones are essential in this endocrinological system for controlling pregnancy and delivery. The placenta is an important temporary endocrine organ in addition to its role in transporting substances between the mother and the fetus. As in many ungulate species, the bovine placenta produces significant amounts of estrogens. The site of estrogen synthesis is the fetal portion of the placentome, the cotyledons, where the key enzyme in estrogen synthesis, aromatase (CYP19A1), is localized in the trophoblast giant cells (Schuler et al., 2006; Schuler et al., 2008). In the period close to parturition, pregnancy‐associated estrogens are associated with the softening of the soft birth pathway and stimulation of myometrial activity (Smith et al., 1973; Schams et al., 2003; Schuler, 2000). However, placental estrogen production in cattle is detectable at the early stages of gestation. Placental estrogens are detectable in amniotic fluid as early as around day 50, 3–4 weeks days after the onset of placentation (Eley et al., 1979). As yet, there is little reliable information on the significance of placental estrogens in the early and middle stages of pregnancy. They presumably serve as a placental or uterine growth and differentiation factor (Schuler et al., 2008; Schuler et al., 2018). In favor of a predominantly local function of placental estrogens in the gravid uterus is the fact that in systemic maternal blood, they circulate predominantly as conjugated forms, that is, as sulfates or glucuronides, with oestrone sulfate being the major estrogen from a quantitative point of view (Hoffmann et al., 1997; Schuler, 2021). Sulphation of placental estrogens apparently already occurs predominantly in the trophoblast, where the estrogen‐specific sulphotransferase SULT1E1 is localized in placentomes mainly in the mononuclear trophoblast cells (Khatri et al., 2011). Oestrone sulfate is measurable in the maternal blood of gravid cattle from about day 120–150 of gestation and increases steadily until late gravidity. A greater increase in free estrogens, predominantly estrone, is not observed until the last month of gestation. The concentrations of estradiol‐17ß, which is biologically much more potent, are comparatively much lower (Hoffmann et al., 1997), with a considerable proportion apparently originating in the udder (Janowski et al., 2002). Possibly, however, the udder is not capable of de novo synthesis of E2 from cholesterol, but may depend in this respect on the supply of precursors from the placenta. The bovine placenta can also produce P4. However, the biological necessity of an auxiliary/placental minor P4 source, which becomes evident after the 240th day of pregnancy is still elusive (Hoffmann et al., 1979; Hoffmann & Schuler, 2002; Schuler et al., 1994; Schuler et al., 2006; Schuler, 2000). In late pregnant or intrapartum cattle, uterine torsion can endanger the life of the offspring and may lead to severe degenerative and inflammatory lesions in the uterus (Sickinger et al., 2020). Impairment of uterine perfusion can develop in the uterus depending on the degree of rotation and the duration of the torsion. Because the veins are primarily affected by vascular twisting and constriction, uterine congestion and cyanosis are common. If therapy is not provided promptly, the vessels will thrombose and the damaged uterus will be cut off from the bloodstream, leading to substantial tissue changes in the uterine wall. The fetus's supply of nutrients and oxygen as well as the elimination of CO_2_ and metabolic end products is reduced. In fatal cases, the fetus subsequently dies as a result of intrauterine respiratory and metabolic acidosis (Busch, 1993; Klein & Wehrend, 2006; Rosenberg & Berchtold, 1978). In addition to the uterus and fetus, the placenta may also be affected by the condition. Many researchers have explored the etiology (Schönfelder & Sobiraj, 2005), diagnostic methods, pathogenesis (Erteld et al., 2012) treatment options (Erteld et al., 2014; Klaus‐Halla et al., 2018), hematological/ biochemical values (Schönfelder et al., 2006; Schönfelder et al., 2007; Sickinger et al., 2018) also inflammation biomarkers (CRP) (Schönfelder et al., 2009) of torsio uteri in cattle in details. As a result of torsio uteri, despite successful treatment, an inadequately opened cervix and placental retention often develop. The causes are unclear and have so far been explained by mechanical damage to the tissue (Klein & Wehrend, 2006). To the best of our knowledge, no studies have been conducted to date on the potential consequences of uterine torsion‐ related changes on placental steroidogenesis in late pregnant or intrapartum cows. In addition to estrogens, maternal P4 concentrations were also assessed to determine whether luteolysis had already occurred in UT animals. The aim of our study was to investigate the extent to which intrapartum UT affects the steroid synthesizing capacity of the bovine placenta or the transfer of placental hormones into the maternal circulation. The findings of this study may support the endocrinological clinical diagnostic of intrapartum uterine torsion caused by multiple etiological variables as well.

## 2 MATERIALS AND METHODS

### 2.1 Animals

The study included a total number of 37 intrapartum dairy cows of various breeds (German Brown Swiss, German Holsteins, and German Fleckvieh) and ages. All these animals were housed on farms in Germany's Hesse region before being transported to our clinic by their owners. The uterine torsion (UT) group consisted of 20 animals. The following conditions were considered to indicate that the animal was already in the process of parturition at the time of referral: Expiration of a physiological gestation period if the date of insemination/mating was known, development of the udder, slackening of the broad pelvic ligaments, and vulva edematisation. The presence of UT was verified during vaginal and transrectal examination (Erteld et al., 2012), which also determined the direction and degree of uterine torsion. The time from the diagnosis of delayed calving until the retorsion of the uterus was defined as the duration of UT, whereas the degree of torsion (180°–360°) was detected during therapy. The following conditions had to be met for inclusion in the UT group: there should be no attempts of retorsion before referral to the clinic, maintained standing ability, and rectal body temperature below 39.0°C (Schönfelder et al., 2006). The comparison (C) group included 17 animals in which calving was spontaneous or could be terminated with moderate obstetrical assistance (simple fetal extraction). Examination of these animals showed no problems with their general health and genital organs.

### 2.2 Blood sample collection

In all animals, blood samples were collected approximately 3–5 h following the onset of dystocia suspicion. The use of the blood samples was approved by the local ethics authority through the animal welfare office of our university (internal correspondence number, IRB number: kTV 11–2018). Blood samples from UT and C animals were collected immediately after the initial examination and prior to any obstetrical measures for radioimmunological measurement of estradiol‐17 β (E2), free total estrogen (FTE), conjugated total estrogen (CTE) and progesterone (P4). Blood samples were taken by puncturing the external jugular vein. The samples were allowed to clot and were then centrifuged for 20 min at about 1000 × *g*. The samples were kept at −80°C until they were analyzed.

### 2.3 Radioimmunological determinations

Established and validated radioimmunological methods (Hoffmann et al., 1973; Hoffmann et al., 1992; Hoffmann et al., 1996; Hoffmann, 1976; Richter‐Hanauer et al., 1988) were applied to determine P4, E2, FTE, and CTE in maternal blood plasma. The antiserum used exhibited the following cross‐reactivity: P4: 100%, pregnenolone: 0.69%, 17α‐OH‐P4: 0.49%, testosterone: 0.37%; androstenedione, estradiol‐17β, and between oestrone and cortisol: < 0.01%. To overcome possible matrix effects, samples were extracted twice with hexane before radioimmunological determination. The minimum detectable concentration was 0.1 ng/mL. The intra‐ and interassay coefficients of variation (CVs) were 8.8% and 8.9%, respectively. The radioimmunological measurement of E2 concentrations was performed by a sequential assay as previously described (Hoffmann et al., 1992). Toluene was used for sample extraction; the antiserum exhibited the following cross‐reactivity: E2: 100%, oestrone: 1.30%; androstenedione, cortisol, dehydroepiandrosterone, pregnenolone, P4, testosterone: < 0.01%. The minimum detectable concentration was 2 pg/mL. Intra‐ and interassay CV were 7.1% and 17.6%, respectively. The assay applied for the measurement of free and conjugated total estrogens followed in principle the procedure described in detail by Hoffmann et al. (1996) for the measurement of free and conjugated oestrone. The identical radioimmunological method was used for the determination of free and conjugated estrogens. Different from the cited work, in which a specific antiserum against oestrone was applied, in this study, an antiserum generated against 17β‐estradiol‐17‐hemisuccinate‐BSA was used, which exhibits virtually identical cross‐reactivity against oestrone, estradiol‐17α and estradiol‐17β (Hoffmann, 1976). The separate determination of the free and conjugated forms was achieved by extracting and measuring the free estrogens. Then the sample residue was enzymatically hydrolyzed, extracted again, and the conjugated forms were subsequently determined via the ‘detour’ of the corresponding free forms. The measurement was performed on an estrone standard curve.

### 2.4 Statistical analyses

In this study, the group's UT and C were compared with respect to the variables P4, FTE, CTE, and E2. Before group comparisons, Shapiro–Wilk (SW) test and *F*‐test were conducted to test the normality of the data and homogeneity of variances (HoV), respectively. Normal distribution and homogeneity of variances were present only for E2 concentrations. Therefore, the two‐sample *t*‐test was used for this group comparison. For the remaining group comparisons, the nonparametric Wilcoxon Rank‐Sum test was applied. Also, correlation analysis between the variables within groups was done using Pearson's correlation (ρ) or Kendall's tau (*τ*) correlation coefficients depending on the situation where the data do not follow a normal distribution. All calculations were carried out using the software environment R (R Core Team, 2020), including ‘ggplot2’ and ‘ggpubr’ packages, and the significance level (α) was taken to be 0.05 in the statistical tests.

## 3 RESULTS

**FIGURE 1:**
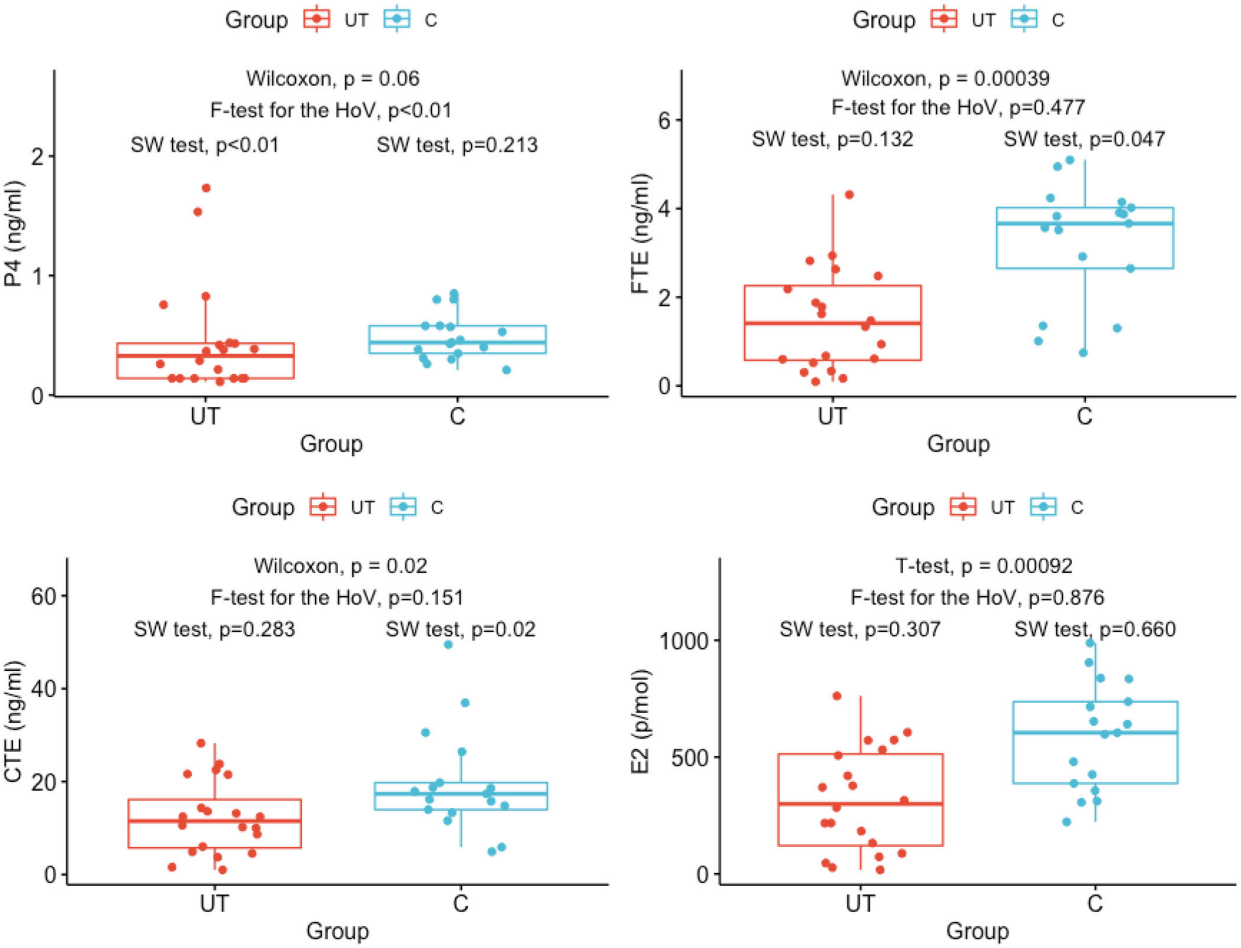
Blood P4, FTE, CTE, and E2 concentrations in UT and C groups. The box's lower and upper borderlines indicate the first and third quartiles, respectively. The horizontal line crossing the box shows the median of the data. Points below or above the boundary of the vertical line represent possible outliers. In terms of P4, there is no statistical difference between the two groups. All estrogen hormones, however, were significantly lower in the UT group than in the C group. E2: estradiol‐17β, FTE: free total oestrogen, CTE: conjugated total oestrogen, P4: progesterone.

**FIGURE 2:**
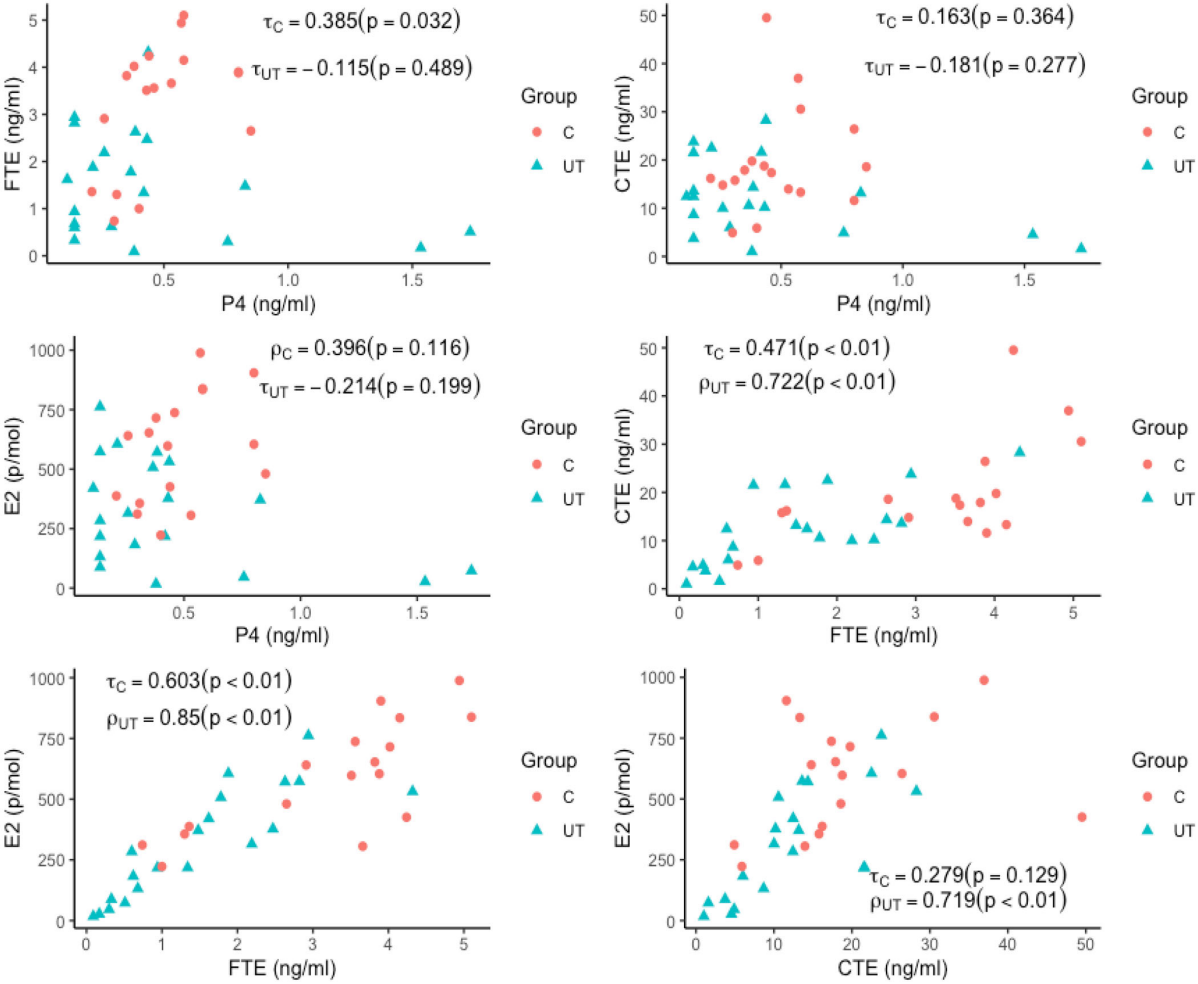
Steroid hormone correlation analyses in the UT and C groups. E2: estradiol‐17β, FTE: free total oestrogen, CTE: conjugated total oestrogen, P4: progesterone, ρ: Pearson's correlation coefficient, τ: Kendall's correlation coefficient.

The results are summarised in Figures 1 and 2. The P4 concentrations in the UT and C groups were not statistically different (*p* = 0.06). Conversely, peripheral FTE (*p* = 0.00039), CTE (*p* = 0.02), and E2 (*p* = 0.00092) levels in the UT group were significantly lower when compared to the C group (Figure 1). Correlation analyses yielded significant positive correlations between FTE and CTE in the UT (*p* < 0.01) and the C (*p* < 0.01) groups (Figure 2). Also, there was significant positive correlation between FTE and E2 in the UT (*p* < 0.01) and C (*p* < 0.01) groups (Figure 2). A significant positive correlation between CTE and E2 (*p* < 0.01) was only found in the UT group but not in C animals. In the C group, there was a significant positive correlation between FTE and P4 (*p* < 0.05) (Figure 2).

## 4 DISCUSSION

Consistent with our own hypothesis, the UT‐induced impairment of placental perfusion and placental function is evident from significantly reduced estrogen concentrations. Here, the difference between the UT and C groups as measured by the *p‐* value from the statistical evaluation is more pronounced for E2 and FTE than for CTE. This is possibly due to the fact that the sulfated steroids have a longer half‐life than the free estrogens due to binding to albumin (Schuler, 2021), whereby the degree of impairment of oestrogen production or delivery to the maternal compartment is reflected in a more current form in the case of the free estrogens than by the conjugated estrogens. Decreased availability of placental estrogens or other placental regulatory factors in the maternal compartment could also lead to impaired uterine function in the subsequent puerperium even in cases of successful obstetric therapy. In addition to impaired production of placental estrogens or their transfer to the maternal compartment due to the disturbance of uterine perfusion, it would also be conceivable that a primary disturbance of placental estrogen and prostaglandin production, via impairment of myometrial activity, could favor the occurrence of uterine torsion.

From a clinical point of view, the differentiation of a prepartum UT from an intrapartum UT is essentially based on the presence of signs of a birth that has already commenced, but which cannot always be clearly assessed by the effects of UT, such as the degree of opening of the cervix or myometrial activity. From an endocrinological point of view, in cattle, the onset of parturition is characterized by the initiation of prepartum luteolysis, which is responsible for the prepartum drop in maternal P4 concentration to basal levels (Schuler et al., 2018). In two cases, clearly, suprabasal levels were measured in UT animals despite the clinical diagnosis of intrapartum UT, suggesting that luteolysis had not yet commenced or at least had not yet been completed in them. However, in animals with intrapartum UT, a basal progesterone level need not necessarily be due to physiological luteolysis, because in severe cases luteal function could also have been terminated by pathological processes.

In the present work, a significant positive correlation was found between P4 and FTE in group C. This observation is difficult to explain. Although the bovine placenta exhibits significant P4 concentrations in placental tissue from local production from approximately 6 months of gestation (Tsumagari et al., 1994), by far the majority of P4 measurable in the peripheral maternal circulation is always of ovarian origin throughout gestation (Schuler et al., 2008). In the final phase of gravidity, no contribution of the bovine uterus to maternal progesterone levels was detectable (Comline et al., 1974). Thus, it is unclear how a relationship between the basal or slightly suprabasal P4 concentrations at birth and the highly variable concentrations of free estrogens could be established. In any case, as a precursor of estrogen production, P4 does not play a quantitatively significant role in cattle and other ruminants due to the minimal lyase activity of ruminant CYP17A1 in the Δ4‐pathway of steroidogenesis (Schuler et al., 1994; Shet et al., 2007).

## 5 CONCLUSION

The current study's findings confirm that intrapartum UT in cows clearly has an adverse influence on the production of placental oestrogens and/or on their release into the maternal circulation, considerably decreasing peripheral levels of estrogen. Significant involvement of the placenta in E2 synthesis is also supported by the fact that in UT cases E2 concentrations are considerably reduced. Further studies are needed on the relationship between the angle of uterine rotation and the reduction in maternal estrogen concentrations. In addition, this study has the following limitations. Blood samples were taken only once during parturition, and there are no data on hormone concentrations before uterine torsion. The animals belonged to different breeds and were of different ages. The degree of uterine torsion varied. In some animals, a retorsion attempt was probably made before transfer to the clinic. Continued studies should clarify the possible effects of each of these factors.

## AUTHOR CONTRIBUTIONS

Sait Sendag: conceptualization, methodology, validation, investigation, writing – original draft. Marlene Sickinger: investigation, methodology, writing – review & editing. Talha Arslan: statistical results. Gerhard Schuler: conceptualization, methodology, validation, writing – original draft, supervision. Axel Wehrend: writing – review & editing, supervision.

## CONFLICT OF INTEREST STATEMENT

The authors declare that they have no competing interests.

## FUNDING

This study did not utilize any grant funds.

### ETHICS STATEMENT

The use of the blood samples was approved by the local ethics authority through the animal welfare office of the Justus Liebig University Giessen (internal correspondence number, IRB number: kTV 11‐2018).

### PEER REVIEW

The peer review history for this article is available at https://publons.com/publon/10.1002/vms3.1145.

## Data Availability

The data presented in this study are available on request from the corresponding author.
